# Beyond Viral Suppression: Navigating Structural Barriers, Aging and Frailty, Drug Resistance, Therapeutic Innovations, and Reproductive Health Challenges in the Global HIV/AIDS Epidemic

**DOI:** 10.33696/aids.7.067

**Published:** 2025

**Authors:** Ahizechukwu C. Eke, Uzoamaka A. Eke

**Affiliations:** 1 Division of Maternal Fetal Medicine, Department of Gynecology and Obstetrics, Johns Hopkins University School of Medicine, Baltimore, Maryland, USA; 2 Division of Clinical Care and Research, Institute of Human Virology, University of Maryland School of Medicine, Maryland, USA

**Keywords:** HIV, AIDS, Pregnancy, Transcriptomics, Metabolomics, Proteomics, Cabotegravir, Lenacapavir, Frailty, Dolutegravir, HIV and Aging, Therapeutic innovation

## Abstract

HIV programs worldwide have achieved remarkable gains toward viral suppression, transforming HIV from a fatal illness into a chronic condition for many. Despite these successes, a substantial proportion of people living with HIV (PLWH) continue to face poor health outcomes that extend well beyond viral control. Persistent social determinants of health and structural barriers, including poverty, stigma, discrimination, and disrupted health systems, limit access to prevention, treatment, and retention in care. At the same time, demographic shifts have created new challenges, with more than half of PLWH in high-income countries now aged ≥50 years, facing accelerated aging, multi-morbidity, and neurocognitive decline. Emerging drug resistance, both pretreatment and transmitted, threatens the durability of existing regimens, particularly in low- and middle-income countries where routine resistance testing remains limited. In reproductive health, women of childbearing age remain disproportionately affected, with ongoing complexities around antiretroviral selection, adverse pregnancy outcomes, and the long-term health of HIV-exposed but uninfected (HEU) infants. This narrative review synthesizes evidence from peer-reviewed studies, global guidelines, and major clinical trials published up until September 2025 to explore these converging issues. By highlighting gaps and opportunities across aging, structural barriers to care, social determinants of health, therapeutic innovation, and reproductive health, we underscore the need for inclusive, multidisciplinary, and evidence-based HIV care models for the next decade.

## Introduction

Since its emergence 4 decades ago, over 88 million people have become infected with HIV and nearly half (42 million people) have died of AIDS related illness globally [1]. As of 2023, new infections have reduced by 60% (1.3 million) compared to its peak of 3.3 million in 1995, driven by multifaceted interventions such as the inception of highly active antiretroviral therapy in 1996, prevention of maternal to child transmission in 1998, expanded access to HIV testing in 2007, and the use of antiretroviral therapy (ART) as prevention around 2011. Global initiatives like the establishment of the joint United Nations Program on HIV/AIDS (UNAIDS) in 1996, and the President’s Emergency Plan for AIDS Relief (PEPFAR) in 2003 have facilitated some of these interventions [2–9]. By far, the most impactful of these interventions is treatment as prevention which has established that the risk of HIV transmission among serodiscordant virally undetectable heterosexual or gay couples is zero, leading to the launching of the Undetectable = Untransmittable (U=U) movement by the Prevention Access Campaign (PAC) in 2016, which has over 1,000 partners in 105 countries and the scaling of U=U by the CDC [10,11].

Despite these considerable strides made with diagnosis, ART and prevention, HIV/AIDS remains a global health crisis with nearly 40 million people living with HIV (PLWH) worldwide as of the end of 2023, and 630,000 deaths annually [12]. While ART has transformed HIV infection from a fatal illness to a chronic condition where people can attain a normal quality of life, the complexities and impact of the epidemic continue to evolve both locally and globally. Sub-Saharan Africa remains the most impacted, comprising over two-thirds of PLWH, where young 15–24-year-old women are disproportionately affected, with over 60% of the new infections occurring in this age group. This represents both a public health challenge as well as a unique clinical hurdle as pertaining to pregnancy and post-partum care, given the heightened maternal-fetal risk, altered drug metabolism and immunology associated with pregnancy. In the US, approximately 1.1 million people are living with HIV, 77% of which are men, with over 60% attributed to male-to-male sexual contact, and 64%, people of color [13]. Similar to other high-income countries, over 50% of PLWH in the US are now aged 50 years or older, which highlights the marked improvements in survival but also comes with new challenges related to frailty and other consequences of aging, chronic comorbidities and long-term ART toxicity [14–16]. Hence, these demographic differences highlight the gaps in the response that translate beyond the availability of ART and viral suppression but provide the opportunity to reassess and optimize HIV care across the reproductive, frail, and aging spectrum, as well as color of PLWH across the different continents. This review aims to discuss the contemporary challenges of HIV/AIDS across key epidemiologic patterns of access to care, HIV in pregnancy, frailty and aging in older people with HIV, new therapies and future projections of the epidemic.

## Review Approach

We conducted a narrative review with the goal of synthesizing contemporary evidence on structural barriers, aging and frailty, drug resistance, reproductive and sexual health, and access to HIV care. To identify relevant literature, we developed a broad search strategy using Boolean operators and keywords/Medical Subject Headings (MeSH) related to: “HIV,” “structural barriers,” “social determinants of health,” “aging,” “frailty,” “drug resistance,” “pregnancy”, neonatal and infant HIV care”, “reproductive health,” “and sexual health.” Searches were performed across PubMed/MEDLINE, Embase, the Cochrane Library, Web of Science, Scopus, CINAHL, and Google Scholar, supplemented by the World Health Organization (WHO) and the United States Department of Health and Human Services (DHHS) guidelines and registries of major clinical trials up to September 2025. Reference lists of key articles and reviews were hand-searched to identify additional studies. No language restrictions were applied. Given the narrative nature of this review, inclusion was intentionally broad, encompassing prospective and retrospective cohort studies, cross-sectional analyses, randomized trials, systematic reviews, and meta-analyses that examined the intersection of HIV care with the themes of interest. Studies focusing solely on animal models were excluded. Each included study was critically appraised for relevance, methodological quality, and applicability. Key domains considered were representativeness of the study population, clarity of exposure and outcome measures, adequacy of confounder control, and generalizability to diverse global contexts. We did not conduct formal risk-of-bias scoring; instead, evidence was assessed hierarchically, with well-designed prospective studies and large multi-country cohorts weighted more heavily than small retrospective studies or single-site reports. Systematic reviews and meta-analyses synthesizing high-quality cohorts were given priority where heterogeneity and confounding had been adequately addressed. The narrative approach allowed us to integrate heterogeneous evidence streams and highlight emerging themes. Findings were presented into eight thematic sections: Structural barriers to HIV care; aging and frailty; challenges with ART; promises and pitfalls of therapeutic innovation; HIV and pregnancy; limitations; and an action list of strategies for implementation.

## Access to Care: The Persistent Structural Divide

Access to HIV care remains hampered by persistent social and health inequities, funding and structural barriers in health systems, as well as unprecedented disruptions by wars in regions like Ukraine and the Middle East, and pandemics such as COVID-19 that restrict timely diagnosis, efficient linkage to care, sustained treatment and viral suppression [17–19]. Food insecurity, unemployment, lack of home care and elder support, poor access to transportation, poor mental health care, poor access to preexposure prophylaxis (PrEP), discrimination and systemic racism are among some notable factors limiting access to HIV care [20–22] (Table 1). These factors are especially relevant in minorities who continue to bear the brunt of the HIV epidemic. Hence, it is no surprise that the hardest hit populations are the ones with the poorest access.

In sub-Saharan Africa, the most cited limitation is the distance of up to two hours of travel, poor road conditions and lack of consistent transportation to access care, especially in rural populations. This barrier affects several domains in the HIV care continuum including access to testing, linkage, and retention in care as well as ART adherence and viral suppression due to difficulty picking up medications [23,24]. Additionally, stigma and discrimination inadvertently contribute to the distance barrier where PLWH bypass nearby care centers to travel long distances to access care so that people in their community will not identify them as having HIV infection [25]. In Europe, the ongoing war in Ukraine has disrupted HIV care and over 30% of the WHO European region are virally unsuppressed. In addition, migrants and women are more likely to have delay in HIV diagnosis due to undocumented status, stigma, discrimination, language, and cultural barriers [26,27]. In the US, HIV continues to disproportionately affect Black individuals who, though account for only 13% of the US population, comprise 40% of the new HIV diagnoses and have lower rates of viral suppression compared to Whites [13]. The utilization of PrEP is very poor among women (4%), Blacks (11%, indicated for 40% of population) and Hispanics (13%, indicated for 24% of the population) compared to nearly 70% utilization in Whites where PrEP is indicated in only 26% of the population [28]. This gap in PrEP utilization has been attributed to poor access, education, financial cost, provider bias and medical mistrust, stigma, side effects, among other factors [29,30].

Globally, the 2025 UNAIDS 95-95-95 target (95% know their HIV status, 95% are on ART, and 95% are virally suppressed) towards eliminating HIV by year 2030 is still far from being attained as of 2023 since only 77% of PLWH were receiving ART, 72% had achieved viral suppression and only 86% knew their HIV status [1]. Even in advanced economies like the US, 13% of PLWH are not aware of their HIV status and geographic, racial, gender, religious, cultural, and political disparities remain evident limitations to access, with half of new infections occurring in the rural Southern US [13,21]. Furthermore, HIV clinical trials, care models and comprehensive services for HIV care and prevention often underrepresent or insufficiently serve certain categories of people such as during pregnancy, women, migrant populations, adolescents, transgender people, people who inject drugs and older adults [21,31–33]. Recently, major structural disruptions from the COVID-19 pandemic stretched already tenuous health systems, delaying ART supply chains, routine testing, and maternal HIV services in over 70 countries [34,35]. These setbacks underscore that contemporary HIV care must encompass not only virologic control but also tackle and attain access, equity, and inclusion.

## Aging with HIV: a New Geriatric Paradigm

Aging with HIV has emerged as a new geriatric paradigm as the number of people 50 years and older with HIV continues to increase globally, comprising about a quarter of PLWH, with over 80% living in low and middle income countries, especially Eastern and Southern Africa [36]. Although the number of PLWH older than age 50 is higher in sub-Saharan Africa, the proportion in the US is over 50% and it is estimated to reach over 70% by 2030 in the US and Europe [37,38]. This aging demographic poses a new challenge for HIV management due to the increased risk and complications from comorbidities such as cardiovascular disease, which occur with chronological aging with nearly double increased prevalence in HIV, as well as geriatric syndromes such as frailty, which are also enhanced by both HIV and chronological age [15,39] (Table 2).

Frailty, for instance occurs up to two decades earlier in PLWH compared to their non-HIV infected counterparts [15]. This accelerated aging has been attributed to systemic immune activation due to HIV, which leads to immunosenescence, resulting from a multifaceted interaction of pro-inflammatory cytokines and lymphocyte dysregulation [40,41]. Although ART has been shown to reverse the accelerated epigenetic aging that is seen in untreated HIV, prolonged ART has conversely been implicated in mitochondrial dysfunction, which eventually results in cellular exhaustion, senescence, and apoptosis [42,43]. This mitochondrial dysfunction is not only limited to the original nucleoside analog reverse transcriptase inhibitors (NRTI) but also to the non-nucleoside (NNRTI), protease inhibitors and integrase strand inhibitors [43]. The phenotypic manifestation of this interplay is multimorbidity, falls, disability, institutionalization, alone or in combination, and eventually, death [40,41].

Multimorbidity is higher in OAWH compared to those without HIV, ranging from 25–60% of OAWH including cardiovascular disease, kidney disease, diabetes, dyslipidemia, cognitive impairment, osteoporosis and cancer [44–46]. HIV-Associated Neurocognitive Disorders (HAND), affecting approximately 30–50% of OAWH despite ART is particularly concerning, because of the diagnostic challenges and symptom overlap with age-related dementias such as Alzheimer’s disease and associated morbidity [47–49]. Emerging biomarkers, such as neurofilament light chain (NFL) and glial fibrillary acidic protein (GFAP), are being explored to better understand the neuroinflammatory processes in HAND and may help bypass or supplement the complex neuropsychological testing needed to make the diagnosis [50,51]. In the US, non-AIDS related death occurs in about 47% of PLWH and this number is projected at 70% in PLWH 50 years of age and older [39,52].

Hence, the complexity of HIV-associated aging, comorbidities and geriatric syndromes with their associated high morbidity and mortality requires an urgent, integrated, multidisciplinary engagement of clinical specialties including infectious diseases, geriatrics, cardiology, neurology etcetera, evidence-based screening and monitoring as well as long-term access to care to avert adverse outcomes [38,53]. Interventions hinged on early initiation of ART, ensuring compliance to treatment and maintenance of access to care and treatment of co-infections like Hepatitis C and tuberculosis, which remains the leading cause of death in PLWH globally are imperative in OAWH [54]. Other mediations include tackling cardiovascular, mental, and cognitive health, polypharmacy, bone health, nutrition, exercise and strength building, social support and addressing social determinants of health [15,40,55]. In the monitoring of the 5 goals for improved quality of life (QoL) in OAWH established as a priority by the US National HIV/AIDS Strategy (NHAS) for 2025, out of the five indicators: self-rated health, unmet needs for mental health services, unemployment, unstable housing or homelessness and hunger or food insecurity, only one goal (decreasing hunger by 50%) was met by 2022 [56]. This reinforces the need for continued and concerted efforts to achieve these goals, including the utilization of age-friendly healthcare environments that reduce stigma and promote comprehensive, compassionate care. Globally, there is need for even more collaborative efforts and strategies for caring for OAWH, given the added challenges that are unique to the low-income countries, which have the highest numbers of OAWH and the associated increased morbidity and mortality in these settings [53].

## Challenges with ART

The success of HIV treatment and outcomes over the last 4 decades has been fueled by the advances in the development of highly active ART. Compared to the onset of the epidemic, there are now several safe, highly efficacious ART classes and regimens, many of which have convenient single pill options that are well tolerated by patients. Nevertheless, the challenges of drug resistance, drug-drug interactions and poor tolerability, persist, albeit less common than in the earlier years of the epidemic [57,58]. There is also the burden of committing to taking medication every day, which also poses a challenge in adherence where some patients stop taking their ART because they became tired of taking pills every day [59].

**Figure 1.**
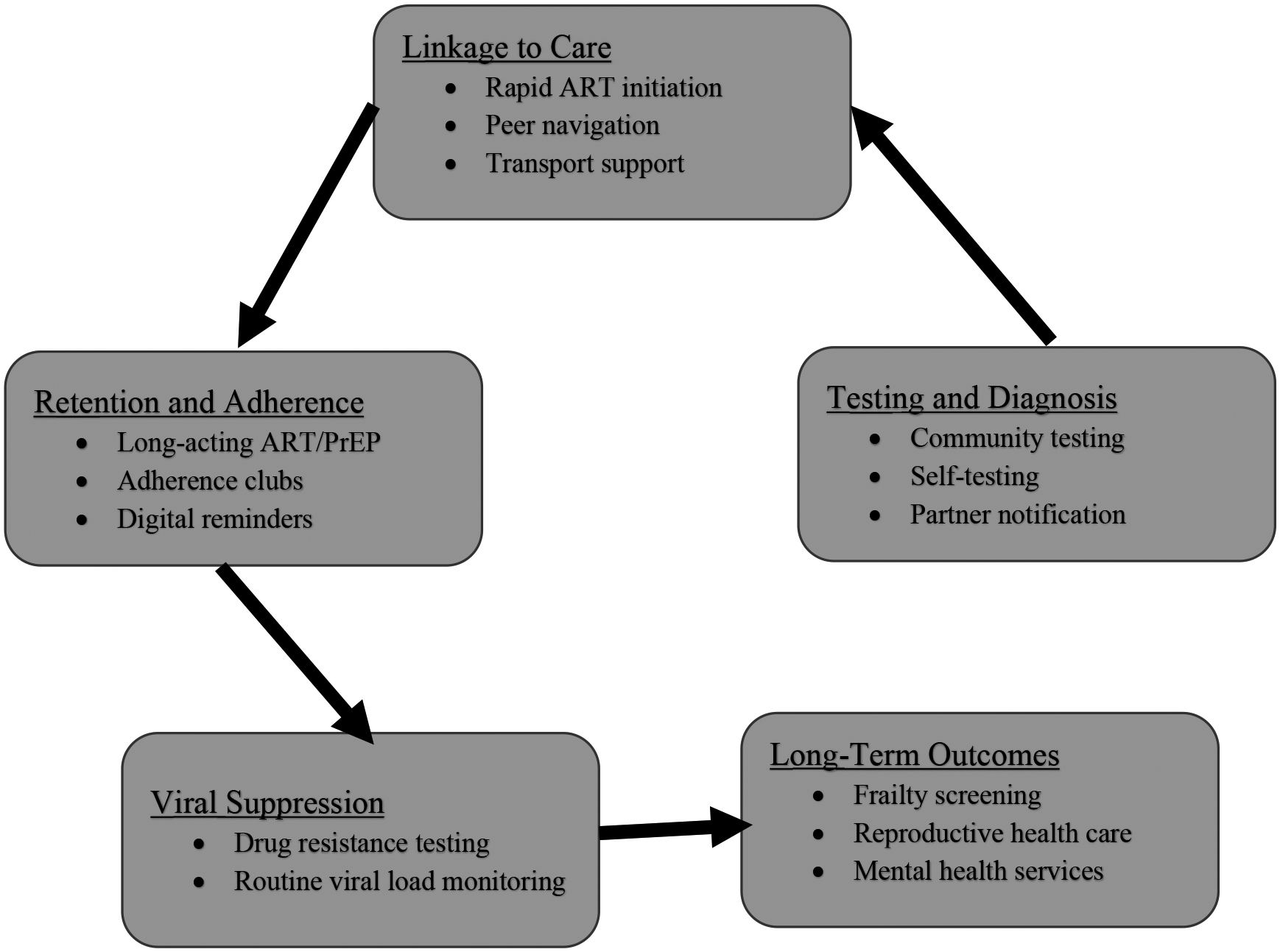
Alignment of key interventions with stages of the HIV patient journey.

With some of these challenges, a new frontier in HIV therapeutics and prevention has emerged, with promising innovations such as long-acting injectable ART and broadly neutralizing antibodies (bNAbs), intravaginal rings for women and dermal implants, as well as curative gene-editing strategies redefining the landscape of viral control and remission (Table 3). Long-acting regimens like cabotegravir-rilpivirine offer the potential to improve adherence and reduce stigma, yet their high cost and cold-chain requirements limit scalability in low- and middle-income countries (LMICs) [60,61]. Clinical trials, such as the First Long-Acting Injectable Regimen (FLAIR) and Antiretroviral Therapy as Long Acting Suppression (ATLAS) studies, demonstrated that this regimen was non-inferior to daily oral ART in maintaining viral suppression among treatment-experienced adults [62,63]. It is exciting that these long-acting agents are also being explored for prevention, with cabotegravir being the first agent approved for PrEP [64]. Another long acting injectable, lenacapavir, a capsid inhibitor, injected subcutaneously every 6 months for people with extensive HIV drug resistance has just been approved for PrEP as of June 2025 requiring injections only twice in the year [65,66]. Lenacapavir received FDA approval in June 2025 for PrEP in at-risk adults and adolescents weighing ≥35 kg [67]. PURPOSE 1 [68] and PURPOSE 2 [66] randomized controlled trials reported that Lenacapavir was superior to daily oral PrEP, with efficacy rates of 100% among cis-gender women and 96% among men and gender-diverse individuals. As these therapeutic innovations accelerate, the biggest concern is cost and accessibility to the populations that need them the most. Access barriers persist, especially in low- and middle-income countries, underscoring the importance of licensing and pricing strategies to ensure global equity. Voluntary licensing and volume guarantee my improve affordability and distribution in low-income countries. Hence, it beckons the important and urgent need for the world health organization, governments, pharmaceuticals, donors and other stake holders to coordinate how to ensure global, ethical and equitable access, prevent disparities, and implement delivery models to accommodate diverse populations, including pregnant women and older adults, gender and racial minorities and LMIC populations. Figure 1 maps key interventions across the HIV care cascade, emphasizing where programs can intervene to improve testing, linkage, retention, and long-term outcomes.

Despite therapeutic advances, ART resistance remains a daunting challenge, especially in resource-limited settings where genotypic testing is not readily available, and key populations such as men who have sex with men, sex workers and people in prison, who have pre-treatment drug resistant (PDR) rates as high as 13–18% [69,70]. PDR to NNRTIs especially is increasing in sub-Saharan Africa, with rates of 23–36% increase per year in East and Southern Africa, which is very concerning since NNRTIs still constitute the first line regimen in some of these regions [69,71]. Hence, the shift to replace NNRTIs with integrase strand inhibitors in these regions. Additionally, transmitted drug resistance (TDR) in new HIV acquisitions has been documented at rates as high as 15% across North America and Europe, with NNRTIs having the highest prevalence, followed by NRTIs and protease inhibitors [72,73]. TDR mutation is uncommon in integrase strand transfer inhibitors, (INSTIs), especially the newer generation INSTIs (<0.8%), making this class of ART likely more suited in an empiric and initial ART regimen before genotype is available [73,74]. It is crucial to take the prevalence and patterns of PDR, including TDR into cognizance to guide treatment choices and potentially prevent the spread of drug resistance because PDR is associated with virologic failure and acquisition of new drug resistance [75–77].

Reassuringly, most cases of high-level resistance to NNRTI is attributed to a small number of resistance mutations, which implies that affordable point-mutation assays may be useful to detect these mutations pre-therapy in regions with high levels of TDR [78,79]. Lack of routine resistance testing potentially leads to unoptimized treatment regimens, treatment failure and clinical progression, which lead to increased risk for adverse outcomes and HIV transmission. Another short fall is that scale up of plasma HIV RNA (viral load) monitoring remains incomplete, especially in LMICs, attributed to logistics such as sample transport and laboratory workflow barriers, finance, and human resources [80,81]. This further complicates HIV management due to the delay in the identification of failing regimens, especially in settings where resistance testing is not readily available. This emphasizes the need for more accessible resistance surveillance programs and viral load monitoring, which could be enhanced by the integration of widespread point-of-care testing across regions. However, the implementation of such testing and the technical requirement would likely be limited by cost amongst other variables.

Drug-drug interactions are not uncommon where treatment of co-infections like tuberculosis (TB) and viral hepatitis B and C are ongoing concurrently or chronic medical conditions requiring medications like statins and proton pump inhibitors. TB remains the leading cause of death among PLWH, with coinfection rates of up to 30% in high-burden countries [82]. Managing TB-HIV co-infection requires coordinated treatment approaches to avoid drug-drug interactions between rifampicin and ART, particularly with protease inhibitors and integrase inhibitors. In addition to drug-drug interactions, Hepatitis B and C co-infections also complicate ART management in other ways such as accelerating liver disease progression, which increases the risk of hepatotoxicity from ART, potentially impacting ART efficacy, and specialized considerations for treatment initiation for ART and regimen selection [83,84]. CMV coinfection, which occurs in up to 90% in PLWH has been linked to persistent systemic inflammation, immune activation, and accelerated immune senescence associated with premature aging as well as increased mortality, even in ART-suppressed individuals [85–87]. Hence, ART optimization must incorporate comprehensive care models that effectively manage co-infections and the drug-drug interactions that complicate HIV treatment in PLWH particularly in regions where healthcare access remains limited.

## Therapeutic Innovation: Promise and Pitfalls

Biologics and immunotherapy represent another frontier in HIV management, with broadly neutralizing antibodies (bNAbs) showing potential promise for long-term viral suppression. These antibodies target conserved epitopes on the HIV envelope, offering potential as both therapeutic and preventive interventions. Recent trials, such as the Antibody Mediated Prevention (AMP) study, demonstrated that the bNAb VRC01 was not effective in preventing HIV acquisition, but may be effective if the virus is susceptible to VRC01 [88]. Further development is focused on combining multiple bNAbs to increase coverage across diverse viral strains. Two recent trials show promise of heterologous bnAB boosting using mRNA germline-targeting vaccine design where a priming immunogen first activates bnAB precursor B cells followed by a series of heterologous boosting immunogens [89,90]. HIV therapeutic vaccines are being developed to enhance HIV-specific immune responses in people already infected with HIV, aiming to reduce viral reservoirs or prevent rebound during ART interruption.

However, challenges remain in inducing robust and durable immune responses, especially in individuals with advanced immunosuppression [91]. Immune-based therapies, while conceptually appealing, must be rigorously tested to avoid unintended immune activation or autoimmune effects, particularly in aging populations where immunosenescence may alter vaccine efficacy.

Efforts to cure HIV have focused on strategies to eliminate latent reservoirs or permanently silence proviral DNA. One approach, known as “shock and kill,” involves reactivating latent HIV using latency-reversing agents (LRAs) followed by immune-mediated clearance (Table 3). Despite the conceptual appeal, clinical trials using histone deacetylase inhibitors (HDACi) or toll-like receptor agonists have shown limited success in reducing reservoir size [92–94]. Ethical and logistical challenges, particularly the risk of gene editing in germline cells, require comprehensive ethical oversight. Furthermore, the feasibility of implementing these technologies in LMICs is hindered by cost and technical logistics and complexity. As research progresses, it is crucial to balance scientific ambition with ethical responsibility, ensuring that innovations do not disproportionately benefit only high-income settings.

## HIV and Pregnancy: Persisting Obstetric and Therapeutic Complexities

HIV disproportionately affects women of reproductive age, particularly in sub-Saharan Africa, where approximately 62% of new infections occur among women in 2023 [95]. Each year, an estimated 1.3 million individuals living with HIV become pregnant, the majority of whom reside in sub-Saharan Africa [96]. In 2024, approximately 84% (range: 72% to >98%) of pregnant women living with HIV had access to antiretroviral therapy to prevent mother-to-child transmission [97]. These regions face significant challenges in addressing maternal morbidity and mortality among women with HIV, as pregnancy can exacerbate the immune compromise associated with HIV [98]. Despite the expansion of ART coverage, maternal deaths remain disproportionately high among women living with HIV, partly due to delayed ART initiation, co-infections (e.g., tuberculosis), and cardiovascular complications. In addition to maternal outcomes, the increased risk of adverse pregnancy outcomes, including preterm birth, low birth weight, and stillbirth, remains a critical public health concern [96] (Table 4). Addressing these challenges requires a comprehensive approach that includes preconception counseling, optimized ART regimens, and enhanced antenatal care tailored to the unique needs of women with HIV.

One of the most significant successes in managing HIV during pregnancy has been the dramatic decline in mother-to-child transmission (MTCT) due to highly effective ART. With appropriate ART adherence, MTCT rates can be reduced to less than 1% [96,98]. However, the selection of ART during pregnancy is complex, balancing efficacy against potential adverse effects. The dolutegravir (DTG) safety debate exemplifies this challenge. Initially, concerns arose regarding a potential increase in neural tube defects (NTDs) when DTG was initiated around conception, leading to cautionary guidance [99]. Subsequent studies, including additional data from the Tsepamo study, demonstrated that the risk was significantly lower than initially reported [100,101]. As a result, DTG has become first-line therapy for pregnant women with HIV due to its superior viral suppression and tolerability. Beyond drug selection, pharmacokinetic changes in pregnancy such as increased volume of distribution and enhanced renal clearance, necessitate close monitoring to maintain therapeutic efficacy [102,103].

Despite viral suppression, pregnancy in women with HIV is associated with a higher risk of maternal-fetal complications. Studies consistently report elevated rates of preeclampsia [104], gestational diabetes mellitus, and preterm delivery [105] compared to HIV-negative counterparts. For instance, a meta-analysis of 28 studies found that women on ART had a higher risk of hypertensive disease of pregnancy compared to those not on ART [104]. Mechanistically, chronic placental inflammation and endothelial dysfunction, potentially exacerbated by both HIV itself and certain ART regimens (e.g., protease inhibitors), may contribute to these outcomes. Furthermore, fetal programming linked to maternal immune activation can have lasting impacts on offspring health, including increased risks of cardiometabolic diseases later in life. The interplay between immune dysregulation and ART exposure during critical developmental windows remains a key area for future research, particularly in understanding the long-term sequelae among HIV-Exposed and Uninfected (HEU) infants.

The inclusion of pregnant women in clinical trials has historically been limited, driven by ethical concerns about fetal risk. As a result, critical gaps persist in evidence-based recommendations for ART safety and dosing during pregnancy (Table 5). Recent policy shifts, including FDA guidance encouraging the inclusion of pregnant women in drug trials and initiatives by networks such as IMPAACT, have continued to address these disparities [106]. For example, the IMPAACT 2010 (VESTED) trial directly assessed the safety and efficacy of INSTI-based regimens compared to Efavirenz-based regimen in pregnant women, offering crucial data that informed global guidelines that has made INSTI central to HIV treatment in pregnancy [107]. Yet, ongoing barriers include liability concerns, regulatory inconsistencies, and the challenge of obtaining informed consent in this complex population [106]. A more inclusive research agenda is essential to bridge these knowledge gaps and ensure that ART regimens recommended for pregnant women are evidence-based and ethically justified.

The health of HEU infants remains a significant concern, as these children are at increased risk for immunological, developmental, and infectious complications. Even when vertical transmission is prevented, HEU infants display altered immune profiles, characterized by chronic inflammation and impaired immune responses, potentially linked to in utero ART exposure and maternal immune activation [108]. For instance, several studies have demonstrated that HEU infants had higher rates of hospitalizations and infectious morbidity compared to HIV-unexposed peers [109,110]. A systematic review comparing cognitive, neurodevelopmental, and behavioral outcomes between older HEU and HUU individuals found that HEU children demonstrated significantly lower performance in at least one domain in a significant number of the included studies [111]. These findings raise concerns about potential long-term functional impairments as the HEU population transitions into adulthood. Addressing these outcomes requires not only vigilant pediatric monitoring but also interdisciplinary care models that include both HIV specialists and developmental pediatricians. Additionally, more robust cohort studies are needed to elucidate the long-term health trajectories of HEU populations, particularly in LMICs where healthcare infrastructure may be inadequate (Table 5).

## Limitations

This review has some limitations. First, because it is a narrative rather than a systematic or scoping review, selection bias may have influenced which studies were prioritized and highlighted despite attempts to include diverse sources. Second, data from certain geographic regions, particularly low-resource settings and from key populations such as adolescents, transgender persons, and older adults remain scarce, limiting the generalizability of findings. Third, heterogeneity in study designs, outcome definitions, and reporting practices across included studies makes direct comparisons challenging and may have led to oversimplification of complex findings. Fourth, many of the interventions discussed—including long-acting agents such as Lenacapavir, community-based service delivery models, and frailty screening approaches, are relatively new, and their long-term effectiveness, cost-effectiveness, and feasibility in real-world programmatic settings are not yet fully established. Finally, although global guidelines were incorporated to provide context, policies and health system capacities vary widely across countries, and the applicability of some recommendations may be constrained by local infrastructure, resources, and sociopolitical environments.

## Conclusion

The evolving landscape of HIV care presents a multifaceted set of challenges that reflect the demographic shifts and scientific advancements of recent decades. As HIV increasingly becomes a chronic condition rather than an acute fatal illness, new complexities arise, particularly in the domains of reproductive health, geriatric care, therapeutic innovation, and structural access barriers. In reproductive health, balancing the efficacy and safety of ART during pregnancy remains a priority, as does addressing the maternal-fetal complications that persist despite viral suppression. The growing cohort of older adults living with HIV underscores the need for integrated care models that address multimorbidity and the unique challenges of aging with a chronic viral infection. Meanwhile, therapeutic innovations such as long-acting antiretrovirals, biologics, and curative strategies offer transformative potential but also demand careful consideration of implementation barriers and equity of access. At the same time, persistent structural divides in HIV care, including disparities by race, geography, and socioeconomic status, continue to undermine global progress in achieving universal access and viral suppression.

Addressing these challenges requires holistic, equitable, and future-facing solutions. Integrating HIV care with non-communicable disease management will be vital as patients live longer and face an increasing burden of age-related conditions. To achieve this, healthcare systems must embrace patient-centered, multidisciplinary models that cater to the evolving needs of PLWH across different regions. In addition, innovations in digital health, such as telemedicine and data-driven care, present opportunities to enhance personalized care and optimize adherence, ensuring that these options are accessible and tailored to diverse populations.

From a research perspective, one of the most pressing priorities in HIV research is to expand inclusion in clinical trials, particularly focusing on pregnant women, adolescents, older adults, and underrepresented minorities. Historically, these populations have been systematically excluded from research due to ethical and safety concerns, resulting in significant gaps in evidence-based care. Addressing these disparities requires regulatory frameworks that mandate inclusion, enhanced community engagement to build trust and participation, and adaptive trial designs that accommodate diverse populations. Initiatives like the IMPAACT network and the NIH Inclusion Across the Lifespan policy are positive steps, but sustained commitment is essential to truly bridge these research gaps.

To sustain progress, there must be ongoing political and financial commitment from governments, global health organizations, and international donors. Programs like PEPFAR and the Global Fund have been instrumental in scaling ART access but face challenges from shifting global health priorities and economic uncertainties. Maintaining momentum requires a commitment to strengthening health systems, fostering local capacity, and addressing the social determinants of health that exacerbate HIV vulnerabilities.

Finally, global solidarity is essential. As new epidemics emerge and geopolitical landscapes shift, the global HIV response must remain focused, resilient, adaptable, and committed to eradicating health inequities. By prioritizing inclusion, innovation, and equity, we can move closer to ending AIDS as a public health threat and ensuring that all individuals, regardless of demographics or geography, benefit from the advancements in HIV care and prevention.

## Action List

Based on the findings of this review, several actionable strategies may strengthen HIV care and improve health outcomes for people living with HIV. These include:

1. *Integrate frailty and co-morbidity testing* - Incorporate annual frailty assessments and routine screening for cardiovascular, metabolic, and neuro-cognitive disorders, especially in patients aged ≥50 years of age. This enables early intervention and tailored care plans.
2. *Scale up resistance testing in high TDR-settings* - Expand access to baseline and ongoing resistance testing in regions with >10% transmitted drug resistance. Coupling this with routine viral load monitoring can optimize regimen selection and reduce treatment failure.
3. *Leverage long-acting therapies* - Accelerate access to injectable PrEP (e.g., Lenacapavir, Cabotegravir) and long-acting ART for patients struggling with adherence. These innovations reduce pill burden and improve retention.
4. *Bundle HEU infant follow-up with immunization visits* - Align routine postnatal HIV-HEU infant monitoring with national immunization schedules. This improves retention, reduces missed visits, and strengthens maternal–child health outcomes.
5. *Expand community-led service delivery* - Support differentiated models such as community ART groups, peer navigators, and mobile clinics to bring care closer to patients and reduce structural barriers.
6. *Prioritize reproductive and women’s health in HIV programs* - Ensure women of childbearing age receive safe ART options, access to contraception, and close monitoring during pregnancy, with longitudinal follow-up of mothers and HEU infants.
7. *Address structural and social determinants of health gaps* - Embed stigma-reduction campaigns, transportation vouchers, and policies that eliminate discriminatory barriers to care. These structural interventions are critical to closing gaps in access and outcomes.

## Figures and Tables

**Table 1.** Key determinants of health affecting HIV care access and outcomes.

Determinant	Impact on HIV care
Food insecurity & poverty (including unemployment and lack of home/elder support)	Limited resources hinder transportation, medication adherence and attendance at appointments; exacerbate disparities in minorities
Transportation barriers & distance to care	Up to two-hour travel times, poor roads and inconsistent transport reduce access to testing, linkage and retention in care and make it difficult to pick up ART
Stigma & discrimination	People travel long distances to avoid being recognized; stigma discourages testing and care engagement
Mental health services & PrEP access	Poor mental-health care and limited access to PrEP reduce comprehensive prevention and treatment options
Racial & ethnic disparities	Black people are 13% of the US population but account for 40% of new diagnoses and have lower viral suppression; women and Hispanics also face poor PrEP uptake [13].
War and conflict (e.g., Ukraine)	Conflict disrupts health services; >30% of PLWH in the WHO European region are not virally suppressed [26,27].
Migrants, undocumented status & gender	Migrants and women face delayed diagnosis due to stigma, discrimination, language and cultural barriers
COVID-19 and other structural disruptions	Pandemic stretched health systems, delaying ART supply chains, routine testing and maternal HIV services in >70 countries [34,35].

**Table 2.** Aging, co-morbidities, and drug resistance challenges in people living with HIV (PLWH).

Metric	Data/Range	Interpretation
PLWH aged ≥50 years (global)	Approximately 25% of PLWH [36].	Majority (>80 %) reside in low- and middle-income countries [36].
PLWH aged ≥50 years (USA)	>50% currently; projected >70% by 2030 [37,38].	Reflects successful treatment but also rising geriatric burden
Frailty onset	Approximately 20 years earlier in PLWH than in HIV-negative peers [15].	Driven by chronic immune activation and immunosenescence
Multimorbidity prevalence in older adults with HIV	Approximately 25–60% [44–46].	Includes cardiovascular, kidney, metabolic and cognitive disorders
HIV-associated neurocognitive disorder (HAND)	Approximately 30–50% prevalence [47–49].	Despite ART, HAND remains common in older PLWH
Non-AIDS-related mortality	Approximately 47% of deaths in PLWH; projected 70% in PLWH aged ≥50 years [39,52].	Reflects shift toward comorbidity-related deaths
Pre-treatment drug resistance (PDR) in key populations	Approximately 13–18% [69,70].	Highest in low- and middle-income countries, men who have sex with men, sex workers and people in prison
Transmitted drug resistance (TDR) in new infections	Approximately 15% across North America & Europe [72,73].	NNRTIs show the highest prevalence of TDR
TDR in new-generation INSTI class	<0.8% [73,74].	Suggests integrase inhibitors are suitable for empiric therapy
TB co-infection rate in high-burden countries	Up to 30% [82].	Tuberculosis remains the leading cause of death in PLWH

**Table 3.** Therapeutic innovations and challenges.

	Therapeutic approach	Potential benefits	Challenges
1	Long-acting ART (e.g., cabotegravir–rilpivirine)	Reduces pill burden; improves adherence; lowers stigma through infrequent injections	High cost; cold-chain storage; risk of resistance with missed injections
2	Broadly neutralizing antibodies (bNAbs)	Provide extended viral suppression; can be used for therapy or prevention	High cost; cold-chain logistics; virus susceptibility issues and need for combination therapy yet to be approved
3	Gene editing (e.g., CCR5 modification)	Potential pathway toward a cure by eliminating HIV reservoirs	Off-target effects; ethical concerns; accessibility and cost barriers in low-resource settings
4	Therapeutic vaccines (heterologous prime-boost or mRNA approaches)	Stimulate robust HIV-specific immunity; may reduce viral reservoirs and prolong remission	Variable efficacy; challenges in inducing durable responses, especially in individuals with advanced immunosuppression
5	Capsid inhibitors (e.g., lenacapavir)	Require injections only twice per year, improving adherence and convenience	High cost; scalability in low- and middle-income countries; need for careful monitoring of resistance
6	Intravaginal rings and dermal implants	Offer female-controlled prevention options; provide sustained drug delivery	Acceptance and adherence issues; potential local irritation; cost and distribution challenges
7	Latency-reversing agents (“shock-and-kill”)	Aim to reactivate latent HIV reservoirs so that infected cells can be cleared, a critical step toward a cure	Limited success of current histone deacetylase inhibitors and toll-like receptor agonists; toxicity and logistical complexity
8	Digital health & telemedicine	Enhance personalized care and adherence through remote monitoring and data-driven interventions	Digital divide and limited technological access in low-resource settings; need for equitable implementation

**Table 4.** HIV and Pregnancy: Key statistics and challenges.

Metric/issue	Data/observation
Annual pregnancies among women with HIV	Approximately 1.3 million pregnancies globally [96].
Women’s share of new infections (2023)	Approximately 62% of new HIV infections occur among women [95].
Pregnant women with HIV receiving ART (2024)	Approximately 84% coverage; range 72%–>98% [97].
Risk of mother-to-child transmission (MTCT) with ART	<1% when ART is adhered to [96,98].
Common maternal complications	Increased risk of preeclampsia, gestational diabetes and preterm delivery
Dolutegravir (DTG) use in pregnancy	Initial neural-tube defect concerns; later studies show lower risk, making DTG first-line therapy
HEU infant outcomes	Higher hospitalizations and infectious morbidity; cognitive and developmental challenges

**Table 5.** Comprehensive challenges and approaches to HIV in pregnancy.

Domain	Key challenges	Underlying issues	Comprehensive approach/strategies
Maternal ART coverage	Not all pregnant women with HIV receive ART (approximately 84% coverage)	Limited access, delays in ART initiation; resource constraints	Scale up ART programs; early diagnosis and linkage to care; strengthen supply chains
Maternal health complications	Elevated risk of preeclampsia, gestational diabetes and preterm delivery	Chronic placental inflammation, endothelial dysfunction; HIV and some ART regimens (e.g., protease inhibitors)	Multidisciplinary antenatal care; monitor blood pressure and glucose; adjust ART regimens; manage co-morbidities
ART selection and pharmacokinetics	Choosing safe, effective regimens; concerns about teratogenicity (e.g., initial DTG neural-tube defect alarm)	Pregnancy changes drug distribution and clearance; risk of drug interactions	Use evidence-based regimens (DTG now first-line); adjust dosages; monitor pharmacokinetics
Mother-to-child transmission & infant outcomes	Residual MTCT risk and adverse infant outcomes; HEU infants have higher hospitalization and cognitive risks	Incomplete adherence, co-infections (e.g., TB), maternal immune activation	Ensure adherence to ART; provide preconception counseling; monitor HEU infants; integrate developmental pediatrics
Research and evidence gaps	Pregnant women historically excluded from clinical trials; paucity of safety and dosing data	Ethical concerns, liability issues, regulatory barriers	Include pregnant women in HIV drug trials (e.g., IMPAACT network); adopt adaptive trial designs; harmonize regulatory frameworks
